# Rare earth ion Tb^3+^ doped natural sodium feldspar (NaAlSi_3_O_8_) Luminescent properties and energy transfer

**DOI:** 10.1038/s41598-019-51171-3

**Published:** 2019-10-10

**Authors:** Dilare Halmurat, Taximaiti Yusufu, Qing-ling Wang, Jiuyang He, Aierken Sidike

**Affiliations:** 1 College of Physics and Electronic Engineering, Xinjiang Normal University, Urumqi, 830054 China; 2 Key Laboratory of Mineral Luminescent Material and Microstructure of Xinjiang, Urumqi, China; 3 Laboratory of Novel Light Source and Micro/Nano-Optical, Xinjiang Normal University, Urumqi, 830054 Xinjiang China

**Keywords:** Chemistry, Materials for optics

## Abstract

In this study, Tb^3+^—doped natural sodium feldspar (NaAlSi_3_O_8_) phosphors have been successfully prepared using high−temperature solid—state method with natural sodium feldspar as a substrate. Energy—dispersive X—ray spectrometry analysis (EDX) of NaAlSi_3_O_8_ showed that 0.03 wt% of Eu element was present, and elemental distribution mapping analysis showed that the distribution of trace Eu in minerals was aggregated. The crystal structure and luminescence properties of the natural sodium Eu—containing feldspar and synthetic sodium feldspar NaAlSi_3_O_8_:Eu^3+^, Tb^3+^ phosphors are discussed in detail. The crystal structure analysis of the samples showed that the Na^+^ in the natural matrix was partly replaced by the doped Tb^3+^. Studies on the photoluminescence properties of the samples indicate that Eu does not form a luminescent center in the natural mineral, however, the strong characteristic peak of Eu^3+^ at 615 nm appears after doping with Tb^3+^ and the peak at 615 nm increases with the increase of Tb^3+^ concentration. According to the above spectral results, the energy transfer from Tb^3+^ to Eu^3+^ is obtained. Through the measurement and analysis of color coordinates, it is found that with the increase of Tb^3+^ concentration, the luminescence color of the samples can be regulated in the green to red region. NaAlSi_3_O_8_:Eu^3+^ Tb^3+^ phosphors has potential application value.

## Introduction

Luminescent materials produced by natural minerals have the advantages of low production cost, simple in preparation and wide applicability. Many researchers at home and abroad have conducted significant research on it. However, natural minerals contain luminescence quenching impurities, which affect the luminescence characteristics of minerals, and become difficult in the field of research. Modern mineralogy shows that these impurities do not all cause luminescence quenching and can be fully utilized. If rare- earth ions can be added into the natural mineral to improve the luminescence properties, the utilization of the natural minerals can be significantly improved. The most abundant of earth minerals is silicate minerals. The structures of a variety of silicates and metal cation bonds impart silicate-based rare-earth luminescent materials with good chemical stability and thermal stability, therefore, become a widely used luminescent material matrix^1^. The minerals of the feldspar mainly include K [AlSi_3_O_8_]-Na [AlSi_3_O_8_]-Ca [Al_2_Si_2_O_8_]. Potassium-sodium feldspar (alkaline feldspar) is widely used in the luminescence measurement technology. Jeak I *et al*. pointed out that there is a strong special emission band in the feldspar doped with Ga, In and Ti, such that the luminous intensity is increased by more than 100 times^2^. Ademola J A *et al*. found that the lowest excited state of the impurity center of the alkaline feldspar is related to the excited state of P, and the higher excited state is related to oxygen^3^. Poolton N R J have also found that many optical transitions can occur in the band gap and sub-band gap excitation regions of the alkali feldspar. It has been reported that the luminescence method utilizing a synchrotron can potentially provide several means to firmly establish the all-optical characteristics of the naturally produced broad-band gap luminescence system, which is essential for the development of its radiation dosimetric properties^4^. In 2017, Hairegu T *et al*. studied the luminescent properties of Sm^3+^ doped natural albite phosphors and obtained high-quality fluorescent materials with high purity in the orange-red region. The mineral albite used in this work contains a small amount of Eu, however it can not be used as a luminescent center in the natural state. It is well known that the energy transfer of Tb^3+^ to Eu^3+^ is very effective because their energy level distributions have a large overlap. We use this feature to achieve the energy transfer of Tb → Eu by doping Tb into albite, which causes the luminescence of Eu. By changing the concentration of terbium, phosphors emitting different fluorescent colors are obtained.

## Results and Discussion

### SEM-EDX analysis of natural sodium feldspar (NaAlSi_3_O_8_)

**Figure 1:**
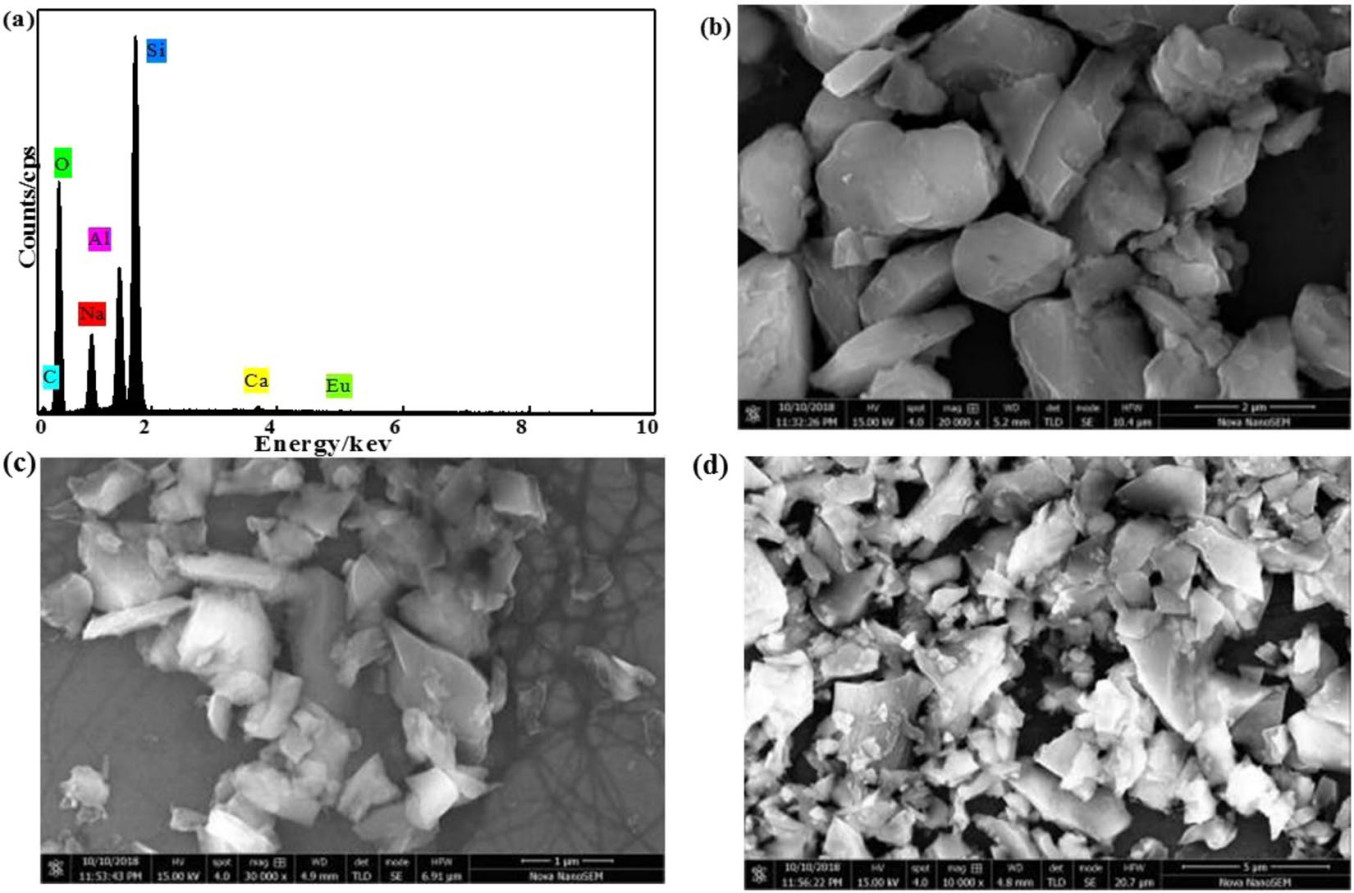
(**a**) EDX patterns of natural sodium feldspar, SEM images of natural sodium feldspar (**b**) thermally-treated natural NaAlSi_3_O_8_: Eu (**c**) and NaAlSi_3_O_8_: Eu, 3%Tb^3+^ (**d**).

Energy-dispersive X-ray spectrometry (EDX) is a standard procedure for identifying and measuring the constituent elements of the samples. The minerals used in this experiment were analyzed by the EDX. Figure 1(a), Table S1 show the EDX analysis report of the natural sodium feldspar.

Figure 1(a) and Table S1 showed that the natural matrix albite in this experiment contained small amounts of C, Ca, and Eu elements, except the main elements Na, Al, Si, O. The atomic percentage of the main elements is 0.85:1:2.6:8.3, close to the atomic percentage of albite 1:1:3:8, and the percentage of the weight of the Eu element was 0.03%. Figure S1 shows element distribution mapping of natural sodium feldspar, Na, Al, Si, O and a small amount of impurity Ca and Eu as impurity can be seen evenly in the map. However, the distribution density of the trace component Eu is uneven, which is concentrated in a certain part of the natural sodium feldspar. Therefore, assumed that Eu in natural minerals may not form luminescent center. Figure S2 shows element distribution mapping of thermally-treated natural sodium feldspar, after treatment, Eu elements are slightly dispersed. In the following passage, natural sodium feldspar in this experiment will be denoted by natural NaAlSi_3_O_8_:Eu^3+^.

In order to get deep insights into the natural sodium feldspar, the surface states of phosphor particles were analyzed in a detail. Figure 1(b–d) displays the surface morphology of natural sodium feldspar, thermally-treated natural NaAlSi_3_O_8_: Eu and NaAlSi_3_O_8_: Eu, 3%Tb^3+^ respectively, it can be seen that samples are irregular particles. The sample before thermally-treated shows an irregular morphology with smooth surfaces. Contrast, the surface of thermally-treated sample seems to be etched and molten, and some small particles are observed, which are initially assumed to be the corrosion products falling off from the natural sodium feldspar particles during the treatment process

### Crystal structure analysis

**Figure 2:**
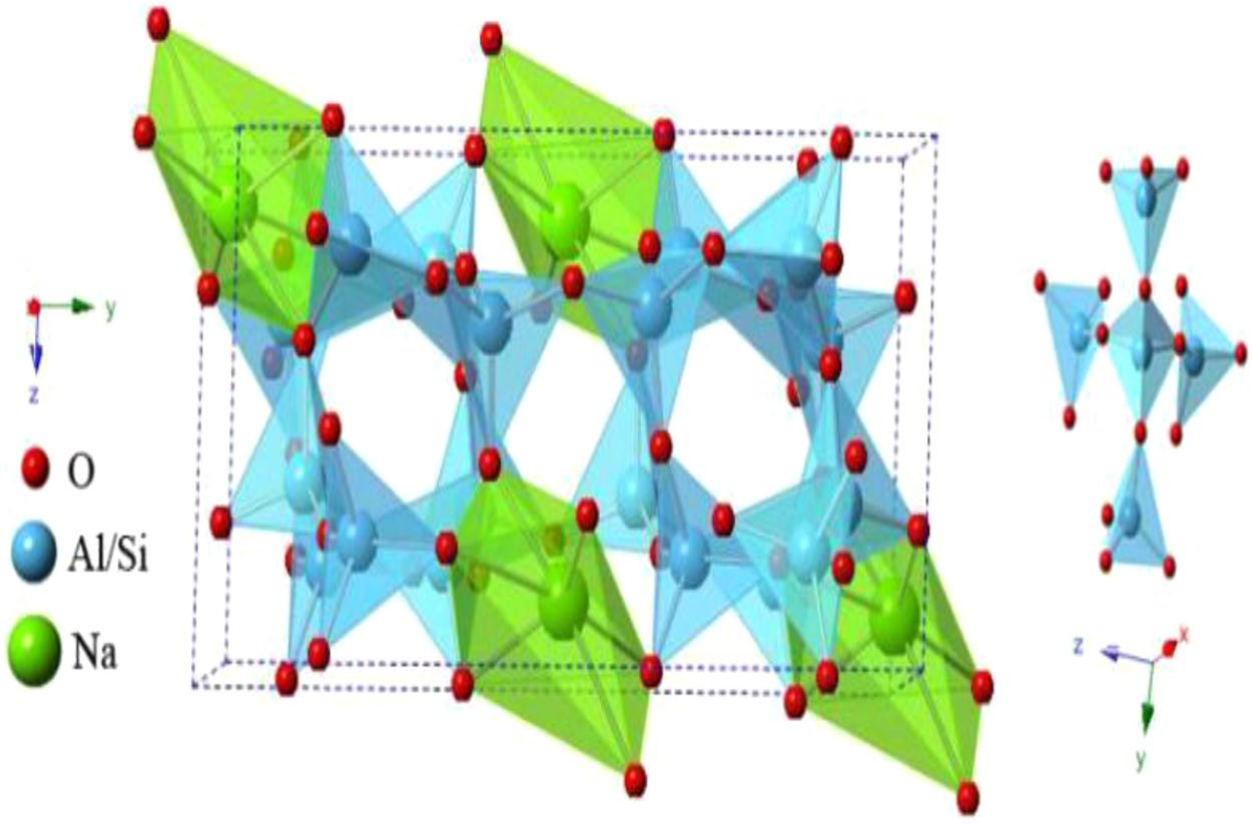
Crystal structure of NaAlSi_3_O_8_ unit cell viewed in the z-direction

Figure 2 shows the crystal structure of NaAlSi_3_O_8_ unit cell viewed in the z-direction. The four corners of each [SiO_4_] tetrahedron are all shared with four adjacent [SiO_4_] to forma Si-O frame structure in oxides, such as quartz. Because the Si in some tetrahedra is replaced by Al, there is excess negative charge, therefore the chemical formula of frame anion is generally written as (Al_x_Si_n−x_)_x_^−^. Because of the existence of the tetrahedral structure, NaAlSi_3_O_8_ has good chemical stability and rigidity^5,6^.

**Figure 3:**
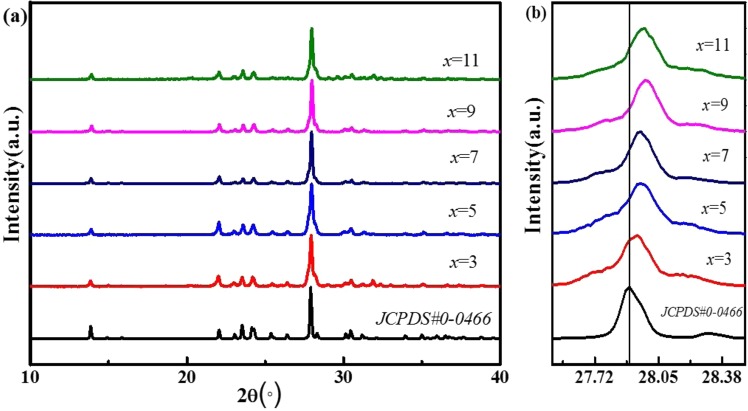
XRD patterns of NaAlSi_3_O_8_:Eu^3+^, x%Tb^3+^.

As shown, the XRD patterns of these samples match well with the standard JCPDS card (No. 9-0466) and no impurity phases were observed, which indicates that slight doping would not lead to a large change in the crystal structure, That is to say, the observed small particles in the thermally-treated sample (Fig. 1b) is either natural sodium feldspar or newly-formed amorphous impurity. The existence of trace elements and increase of the doping concentration of Tb^3+^ lead to a diffraction peak located at 27.9° in 2θ, corresponding to the (002) crystal plane, shifted slightly to a higher angle direction (Fig. 3b), suggesting the substitution of larger Na^+^ (CN = 6, r = 0.102 nm) with smaller Tb^3+^ (CN = 6, r = 0.092 nm). This causes a change in the lattice constant of the host lattice^7,8^. According to the Bragg formula:

$$2\mathrm{dsin}{\rm{\theta }}={\rm{n}}{\rm{\lambda }}$$

The decrease of radius r leads to the decrease of surface spacing d, displacing the peak to a large angle

Figure S3 shows the calculated lattice constants and unit cell volume as a function of Tb^3+^ content. Lattice constants a, b and c calculated by Jade. It is clearly seen that the lattice constants a, b, and c decrease with increasing Tb^3+^ content, and the unit cell volume can be calculated as:

$${\rm{v}}={\rm{abc}}{(1+2\cos {\rm{\alpha }}\cos {\rm{\beta }}\cos {\rm{\gamma }}-{\cos {\rm{\alpha }}}^{2}-{\cos {\rm{\beta }}}^{2}-{\cos {\rm{\gamma }}}^{2})}^{1/2}$$

### Absorption spectrum analysis

**Figure 4:**
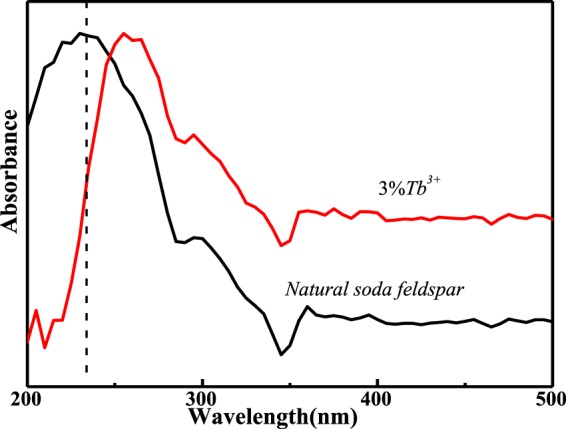
UV-vis absorption spectra of thermally-treated natural NaAlSi_3_O_8_: Eu and NaAlSi_3_O_8_: Eu, x%Tb^3+^(x = 0,3)

Figure 4 exhibits the UV-vis absorption spectra of thermally-treated natural NaAlSi_3_O_8_: Eu and NaAlSi_3_O_8_: Eu, 3%Tb^3+^. At x = 0, the absorption spectra show a strong absorption band around 232 nm. When the Tb^3+^ ions are incorporated into the host lattice, the absorption spectra show a slight red-shift, which indicates that the Tb^3+^ ions are doped successfully into the host lattice, and it is consistent with the XRD results. The cause of the red-shift was already reported by Ahemen·I *et al*. The red-shift of absorption spectra is mainly attributed to the electro-negativity difference between Natrium and Terbium. Since, the electro-negativity of Terbium is higher than that of Natrium (Pauling electro-negativity of Terbium is 1.1 and that of Natrium is 0.98), and thus the electro-negativity difference between Na and O is larger than that of Tb and O. In our case, the introduction of Tb^3+^ may lower the O^2−^ ligand to cations bond energy in comparison with that of pristine sample^9,10^.

### Photoluminescence properties

**Figure 5:**
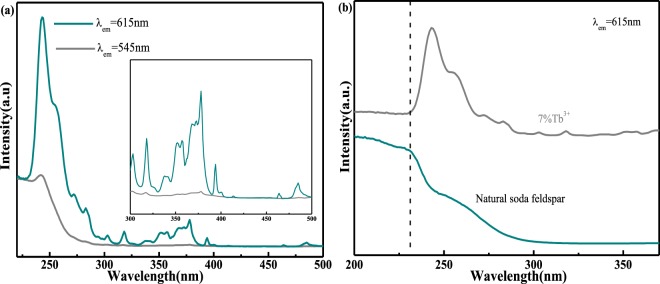
(**a**) PLE spectra of NaAlSi_3_O_8_: Eu, 3%Tb^3+^ (λ_em_ = 545 nm, 704 nm), (**b**) PLE spectra of natural NaAlSi_3_O_8_: Eu and NaAlSi_3_O_8_: Eu, 3%Tb^3+^ (λ_em_ = 615 nm).

Figure 5a shows the PLE spectrum of NaAlSi_3_O_8_ Eu 3%Tb^3+^ monitored at 545 nm, 704 nm. When the monitoring wavelength is 545 nm, a broadband strong excitation band with a peak of 248 nm (220–300 nm) is observed. There are many explanations for the excitation of 220–300 nm containing Eu^3+^ and Tb^3+^ system, such as studies of terbium-europium Co-doped ZrO_2_ system explained that the excitation peaks at 247 nm and 278 nm belong to Eu^3+^-O^2−^ charge transfer zone (CTB) and 4f → 5d transitions of Tb^3+^. In the study of NaGd (MoO_4_)_2_:5% Tb^3+^, 1% Eu^3+^ system, Yao D *et al*. explained that the excitation peaks at 200–300 nm were attributed to the O^2−^ Eu^3+^ and O^2−^ Mo^6+^ transitions^11^. Figure 5b shows the PLE spectrum of natural NaAlSi_3_O_8_: Eu and NaAlSi_3_O_8_: Eu, 3%Tb^3+^ monitored at 615 nm. No excitation peaks were found at the 257 nm, 273 nm, and 284 nm sites of undoped Tb^3+^, and only the 232 nm centric broadband excitation peak was observed. After Tb^3+^ incorporation, the main peak showed a red-shift, and the excitation peak appeared at 257 nm, 273 nm, and 284 nm. Therefore, we determined the broadband strong excitation band (220–300 nm) with the peak of 248 nm in Fig. 5a, which was caused by Eu^3+^-O^2−^ CTB and allowable transition 4f^8^ → 4f^7^5d^1^ of Tb^3+^ Within the range of 300–500 nm, a series of very weak excitation peaks caused by f → f transition of Tb^3+^ ions can be observed. The emission peaks of Tb^3+^ and Eu^3+^ may be located at 589 nm and 613 nm, respectively, in order to exclude the interference from the Tb^3+^ emission, the emission wavelength of 704 nm is selected as the monitoring wavelength, the strong excitation peaks at 248 nm and a series of excitation peaks caused by the superposition of the f → f transition from Tb^3+^ and the 4f → 4f transition of the Eu^3+^ are also observed^12,13^. The excitation peaks corresponding to Tb^3+^ were observed when the Eu^3+^ characteristic emission was monitored. This result confirms that the energy transfer of Tb^3+^ to Eu^3+^ may be effective.

**Figure 6:**
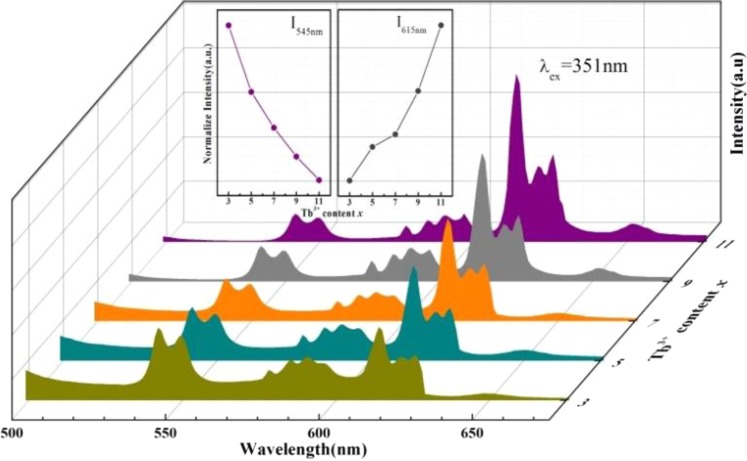
PL spectra of natural NaAlSi_3_O_8_: Eu, x% Tb^3+^(x = 3, 5, 7, 9, 11, λex = 351 nm).

Figure 6 shows the PL (λ_ex_ = 351 nm) spectra of thermally-treated natural NaAlSi_3_O_8_:Eu, x%Tb^3+^ (x = 3, 5, 7, 9, 11). The emission peaks at 545 nm, 586 nm, 591 nm, 621 nm, and 626 nm were observed, corresponding to the ^5^D_4_ → ^7^F_5,4,4,3,3_ transitions of Tb^3+^. The emission peaks at 578 nm 586 nm, 591 nm, 615 nm, 621 nm, 626 nm, 652 nm, correspond to the ^5^D_0_ → ^7^F_0,1,1,2,2,2,3_ transitions of Eu^3+^ ^14^. Therefore, there is a superposition of emission spectra between Tb^3+^ and Eu^3+^ in the range of 586–626 nm. The inset of Fig. 6 shows the concentration dependence of Tb^3+^ luminescence intensity of 545 nm (Tb^3+^) and 615 nm (Eu^3+^). With the increase of Tb^3+^ concentration, the peak at 545 nm decreases, while the peak at 615 nm increases. The concentration of Tb^3+^ increases and the energy is transferred to Eu^3+^ simultaneously. Therefore, the emission peak at 615 nm continues to increase. We also measured the photoluminescence properties of thermally-treated natural NaAlSi_3_O_8_: Eu. Figure S4 shows the PL spectra of thermally-treated natural NaAlSi_3_O_8_:Eu and NaAlSi_3_O_8_:Eu, 3%Tb^3+^. When natural NaAlSi_3_O_8_:Eu absence of Tb^3+^, we observed very weak peaks (Eu^3+^) at 615 nm, 626 nm and 652 nm. Natural sodium feldspar containing Eu does not emit light under ultraviolet lamp, which may be due to low Eu^3+^ content and uneven distribution (Figs S1 and 2). We also measured the quantum efficiency of the sample. When the Tb doping concentration is 3%, 5%, 7%, 9%, 11%, the quantum efficiency is 29%, 33%, 40%, 51%, 67%, respectively.

**Figure 7:**
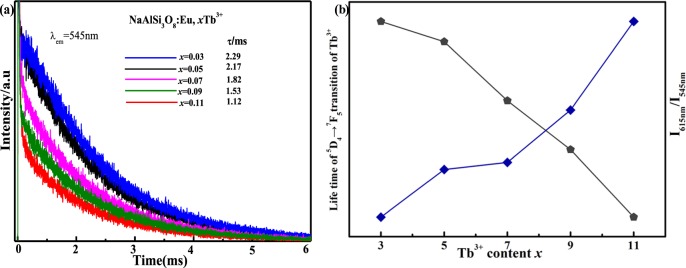
(**a**) Lifetime decay curves for Tb^3+^ in the NaAlSi_3_O_8_: Eu, x% Tb^3+^ phosphors monitored at 545 nm (**b**) concentration-dependent luminescence intensity of 615 nm/545 nm and concentration-dependent lifetime of Tb^3+^.

In order to further confirm the energy transfer mechanism between the Tb^3+^ and the trace element Eu^3+^ in the NaAlSi_3_O_8_: Eu, xTb^3+^ phosphor, it is necessary to test and analyze the transient spectrum in addition to the steady-state spectrum. The fluorescence decay curves of theNaAlSi_3_O_8_: Eu, x%Tb^3+^ sample are shown in Fig. 7(a), calculated by:

$$\tau =\frac{{\int }_{0}^{\infty }I(t)tdt}{{\int }_{0}^{\infty }I(t)dt}$$

where *I (t)* is the luminescence intensity at time *t*. The calculated average lifetimes for different samples are2.29, 2.17, 1.82, 1.53, 1.12 ms, which correspond to the concentrations of x = 3, 5, 7, 9, 11 respectively. The decreased life-time of Tb^3+^ with increased Tb^3+^ concentration demonstrates an effective energy transfer from Tb^3+^ to Eu^3+^. The contributions of the different ions in the corresponding PL spectra have been calculated and are shown in Fig. 7(b). It is obvious that the G/R (green to red) ratio continuously increases^15^.

**Figure 8:**
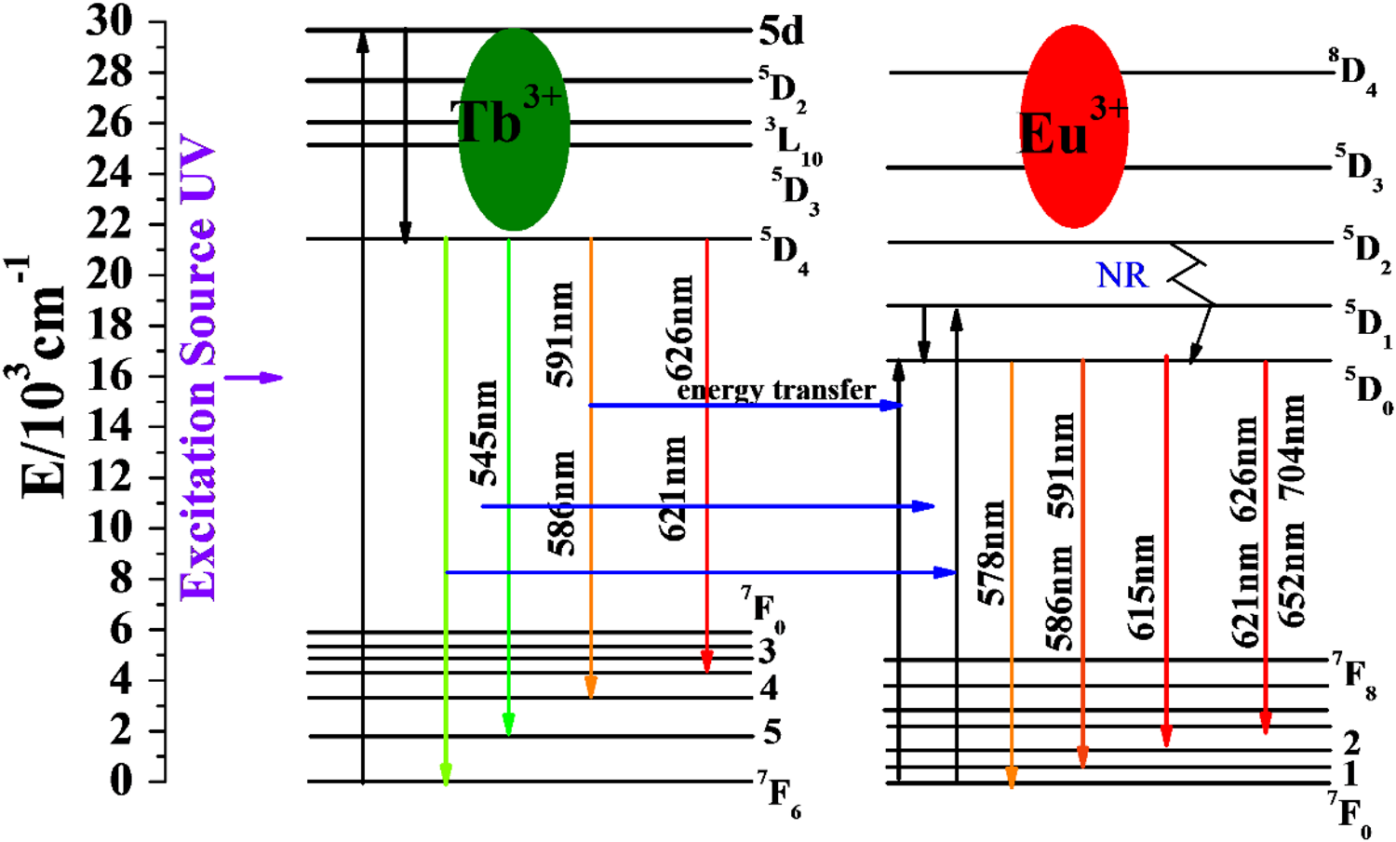
Energy level diagrams of Tb^3+^ and Eu^3+^.

The energy transfer of Tb^3+^ to Eu^3+^ is very effective because their energy level distributions have a large overlap. Figure 8 shows the possible process of energy transfer between Tb^3+^ and Eu^3+^. Under ultraviolet light, the 4f ^8^ electrons of Tb^3+^ transition from the ground state to the excited state 4f^7^5d, then, these electrons relax to the excited state ^5^D_4_ level. Thereafter, the blue-green light (^5^D_4_ → ^7^F_6, 5, 4, 3_) are emitted from the gound state by the polychromatic relaxation, and the energy is transferred to the ^5^D_1_ and ^5^D_0_ levels of Eu^3+^ by cross relaxation. Eu^3+^ absorbs energy from Tb^3+^ and emits orange-red light. The energy transfer of Tb^3+^ to Eu^3+^is effective^16,17^.

### Chromaticity coordinates

**Figure 9:**
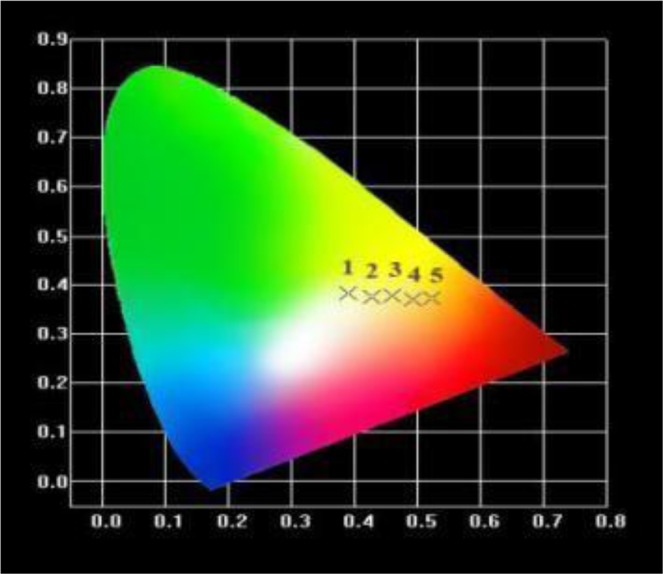
The CIE chromaticity coordinates for NaAlSi_3_O_8_: Eu, x% Tb^3+^ phosphors.

Table 1 and Fig. 9 show the color coordinates and chromaticity diagrams of the NaAlSi_3_O_8_: Eu, x%Tb^3+^ (x = 3, 5, 7, 9, 11) respectively. With the increase of Tb^3+^ concentration, the color coordinates change from (0.3894, 0.3834) to (0.5033, 0.373) which corresponds to the color change of green-yellow to orange under the ultraviolet radiation of 254 nm. It is found that when the concentration of Tb^3+^ is higher, the color of the sample is closer to the red emission (615 nm). The increasing concentration of Tb^3+^ will transfer energy to Eu^3+^, which will increase the emission peak at 615 nm. These results show that the obtained samples exhibit the advantages of polychromatic emission in the visible region and have potential applications in the field of solid illumination.

**Table 1:** CIE chromaticity coordinates of NaAlSi_3_O_8_:Eu, x% Tb^3+^ (x = 3, 5, 7, 9, 11).

Sample no.	x	Excitation (nm)	CIE (x, y)
1	3	254	(3.3894, 0.3834)
2	5	254	(0.4271, 0.37450
3	7	254	(0.4605, 0.3765)
4	9	254	(0.4935, 0.3701)
5	11	254	(0.5233, 0.3730)

## Conclusions

In this study, Tb^3+^ doped natural sodium feldspar (NaAlSi_3_O_8_) phosphors were been successfully prepared by using high-temperature solid-state method with natural sodium feldspar as a substrate. EDX analysis showed that the natural matrix albite in this experiment contained a small amount of C, Ca, and Eu elements except the main elements Na, Al, Si, and O. The percentage of the weight of the Eu element is 0.03%, and the distribution is not uniform. Spectral analysis of phosphors shows that natural albite containing Eu does not form any luminescent centers after heat treatment without Tb^3+^. After doping Tb^3+^, the emission peak 615 nm (Eu^3+^) increases with the increase of Tb^3+^ concentration. The excitation peaks corresponding to Tb^3+^ were observed when the Eu^3+^ characteristic emission was monitored. According to the above spectral results, the energy transfer from Tb^3+^ to Eu^3+^ was obtained. The fluorescence lifetime of the sample confirmed the existence of energy transfer between Tb^3+^ and Eu^3+^. The color coordinate results showed that the sample luminescence can be regulated in the green to red region. Starting from natural materials, a new type of luminescent material is synthesized, which is a feasible and economical way.

## Experimental Procedure

### Preparation of samples

All powder samples of natural sodium feldspar NaAlSi_3_O_8_: x% Tb^3+^ (x% is the mass percentage, x = (3, 5, 7, 9, 11) were synthesized by a high-temperature solid-state method. Natural sodium feldspar and TbF_3_ (99.9%) were well ground in an agate mortar and then shifted to an alumina crucible. After being sintered at 1150 °C for 3 h. All mixtures were collected after natural cooling to room temperature, and pulverized for further measurements.

### Characterization of samples

#### Photoluminescence (PL)

PL spectra were measured at room temperature using a fluorescent spectrophotometer (FL980, Edinburgh, England) equipped with a 450 W Xe light source. The amount of phosphor powder was controlled to be 1.5 g.

#### Decay time

The fluorescence lifetime of the samples was measured by aFLS920 steady-state and transient-state spectrometer and μF920 (EDINBURGHINSTRUMENTS) microsecond pulse flash lamp.

#### SEM-EDS measurements

The surface morphology and the energy-dispersed X-ray spectroscopy (EDS) measurements were performed using a high-resolution field emission scanning electron microscope (FEI, Nova Nano SEM 450) at room temperature.

#### Absorption spectrum

The absorption spectrum of the sample was measured by the Hitachi U-3900/3900H spectrophotometer and the measurement range was 200–500 nm.

#### Powder X-ray diffraction (XRD)

The crystal structures of all the powders were characterized by X-ray powder diffraction (XRD) (Shimadzu XRD-7000) with Cu Kα (λ = 0.15406 nm) radiation at 40 kV and 30 mA at room temperature. The detector covers an angular range 10° < 2θ < 105° with a counting time of 5 s per step.

#### Color coordinate

The color coordinates of the samples were calculated by the CIE software.

## Supplementary information

Rare earth ion Tb3+ doped natural sodium feldspar (NaAlSi3O8) Luminescent properties and energy transfer
