# Supplementary material for: Rare earth ion Tb3+ doped natural sodium feldspar (NaAlSi3O8) Luminescent properties and energy transfer
Source: Sci Rep. 2019 Oct 10;9:14637. doi: 10.1038/s41598-019-51171-3 (PMC6787097; doi:10.1038/s41598-019-51171-3)
Supplement: Supplementary file 1 — Rare earth ion Tb3+ doped natural sodium feldspar (NaAlSi3O8) Luminescent properties and energy transfer [file 41598_2019_51171_MOESM1_ESM.docx]

**Rare earth ion Tb^3+^ doped natural sodium feldspar (NaAlSi_3_O_8_) Luminescent properties and energy transfer**

**Dilare Halimulati ^1, 2^, Taximaiti Yusufu ^1, 2, 3^, Qing-ling Wang ^1 ,2^ , Jiuyang He^1, 2^**

**Aierken Sidike* ^1 , 2, 3^**

^1^ College of Physics and Electronic Engineering, Xinjiang Normal University, Urumqi 830054, China.

^2^ Key Laboratory of Mineral Luminescent Material and Microstructure of Xinjiang.

^3^ Laboratory of Novel Light Source and Micro/Nano -Optical, Xinjiang Normal University, Urumqi 830054, Xinjiang, China

🖂 Aierken Sidike [aierkenjiang@sina.com](mailto:aierkenjiang@sina.com)


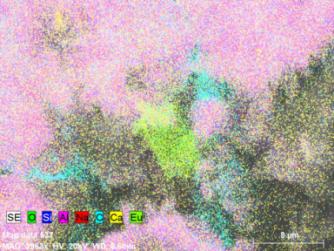


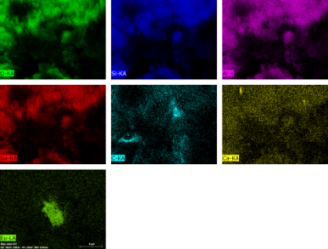


**Fig.S1**  element distribution mapping of natural sodium feldspar


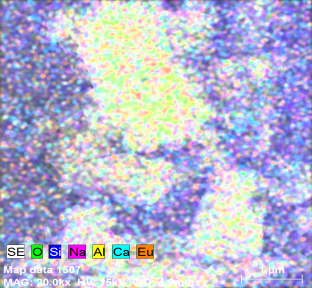


**Fig.S2** element distribution mapping of thermally-treated natural sodium feldspar.


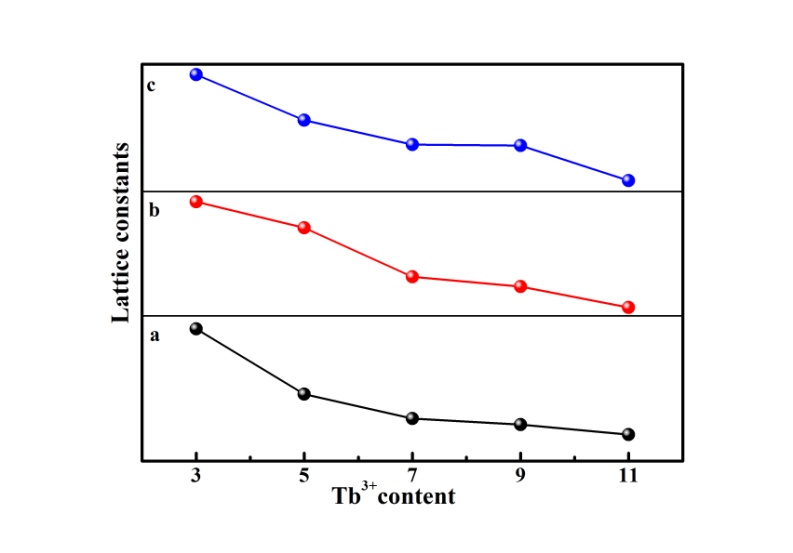

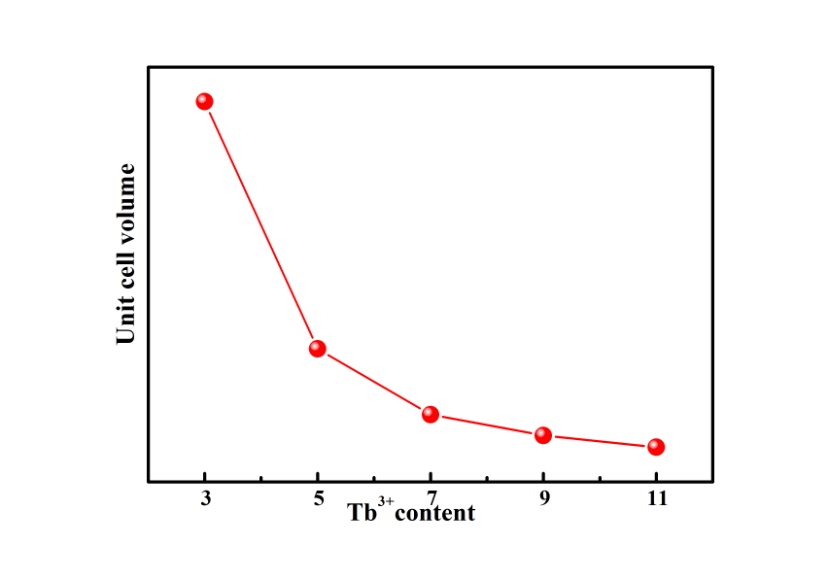


**Fig. S3** Changes in lattice constants and unit cell volumes forNaAlSi_3_O_8_: Eu，x%Tb^3+^ samples.

**Tab. S1** EDX analysis report of Natural [soda](C:/Program%20Files%20(x86)/Youdao/Dict/7.5.2.0/resultui/dict/?keyword=soda)[feldspar](C:/Program%20Files%20(x86)/Youdao/Dict/7.5.2.0/resultui/dict/?keyword=feldspar)

| El | AN | unn . C  [wt.%] | no rm. C  [wt.%] | Atom. C  [a t.%] | (1 Singma)  [wt.%] |
| --- | --- | --- | --- | --- | --- |
| O | 8 | 51.94 | 50.37 | 60.36 | 6.05 |
| Si | 14 | 27.6 | 26.76 | 18.27 | 1.20 |
| Al | 13 | 10.49 | 10.17 | 7.23 | 0.53 |
| Na | 11 | 7.6 | 7.37 | 6.14 | 0.52 |
| C | 6 | 5.05 | 4.90 | 7.82 | 1.08 |
| Ca | 20 | 0.38 | 0.37 | 0.18 | 0.04 |
| Eu | 63 | 0.07 | 0.07 | 0.01 | 0.03 |
